# No theory: an explanation of the lack of consistency in cross-country health care comparisons using non-parametric estimators

**DOI:** 10.1186/s13561-016-0118-2

**Published:** 2016-08-31

**Authors:** Richard Gearhart

**Affiliations:** School of Business and Public Administration, Department of Economics, California State University, 20 BDC, 9001 Stockdale Highway, Bakersfield, CA 93311 USA

**Keywords:** United States healthcare, Cross-country healthcare comparison, Production efficiency, Order- α, OECD, C14, I11, I12, I18

## Abstract

Since 2000 several papers have examined the efficiency of healthcare delivery systems worldwide. These papers have extended the literature using drastically different input and output combinations from one another, with little theoretical or empirical support backing these specifications. Issues arise that many of these inputs and outputs are available for a subset of OECD countries each year. Using a common estimator and the different specifications proposed leads to the result that efficiency rankings across papers can diverge quite significantly, with several countries being highly efficient in one specification and highly inefficient in another. Broad input-output measures that are collected annually provide consistent efficiency rankings across specifications, compared to specifications that utilize specific measures collected infrequently. This paper also finds that broad output measures that are not quality-adjusted, such as life expectancy, seem to be a suitable alternative for infrequently collected quality-adjusted output measures, such as disability adjusted life years.

## Background and literature review

Since the beginning of 2000, a vast literature on cross-country healthcare efficiency comparisons has come into being. Evans et al. [7] and Tandon et al. [24] were the first to utilize parametric estimators and novel healthcare indicators to identify which country healthcare delivery systems were most efficient. Since then, a number of authors have attempted different input-output specifications, using newer parametric and non-parametric estimators, to improve upon the original rankings found in Evans et al. [7] and Tandon et al. [24], and to attempt to guide policy, including on occasion to construct healthcare indices from these rankings.

However, a number of issues with the cross-country healthcare efficiency rankings literature have come into focus. Critics attacked Evans et al. [7] and Tandon et al. [24] on their choice of methodology, the output measures selected by the World Health Organization (WHO), the weights assigned to output measures, assumptions made by researchers collecting the data, and the choice to include Organization for Economic Cooperation and Development (OECD) countries along with non-OECD countries in the sample ([10]; [12]; [16, 27]). Gearhart [8] also noted that there was little correlation between efficiency rankings in the original Evans et al. [7] and Tandon et al. [24] papers and his paper, where he utilized the same input and output measures, but a different, non-parametric, estimator.

This signifies that the literature suffers from a variety of limitations, from methodology, to the choice of estimators, to a lack of data, even in the developed world. An even more disturbing trend is the fact that many of these efficiency ranking studies are run with little theoretical backing for the inputs and outputs that the authors choose to use [1, 2, 4, 9, 11, 22, 23]. The efficiency estimations require inputs and outputs to be chosen, as the healthcare production process turns these inputs into outputs, which allow for efficiency rankings to be inferred. Unfortunately, many of the follow-up papers use rather ad hoc procedures to determine which inputs and outputs should be used. Little has been done to either empirically or theoretically identify which inputs and outputs should be specified as the most important when determining the healthcare production process.

Each of these papers have compelling theoretical reasons for why the individual inputs and outputs should be used, as each impacts health in a different way. However, the authors fail to make a compelling argument as to why these inputs and outputs should be used cohesively, or why they are strict improvements over the efficiency rankings provided in other papers that follow the original WHO studies [7, 8, 10, 12, 16, 24].

As shown in Table 1, since 2005 there have been 9 studies that, at some point, look at efficiency rankings of countries. Of these 9 studies, there are 8 different specifications. Only Gearhart [8] uses a similar specification to that created by the WHO in 2000, though he uses life expectancy rather than a created measure by the WHO, disability adjusted life expectancy (DALE). Another drawback is that, because of differences in collection procedures across countries, not all measures are measured each year. Thus, it may be hard to analyze efficiency changes across years, or analyze productivity improvements (or regression), as some measures are only collected by decade.

**Table 1:** Description of previous papers and the input-output combinations used

Author(s)	Inputs used	Outputs used	Estimator used	Number of countries used	Years used
Adams et al. (2011) [1]	1. Public Spending on Healthcare, % of GDP	1. Transformed Infant Survival Rate (ISR), where ISR is the inverse of the infant mortality rate (IMR), multiplied by the ratio of public spending to total spending on healthcare, both as a % of GDP 2. Transformed Life Expectancy (LE), where life expectancy is multiplied by the ratio of public spending to total spending on healthcare, both as a % of GDP	Input-oriented DEA	19 OECD countries	1980–2000
Afonso & St. Aubyn (2005) [2]	1. Inpatient Beds per 1000 population 2. Number of practicing physicians per 1000 population 3. Number of nurses per 1000 population	1. LE 2. Transformed ISR, where ISR is 1000 minus the IMR, which is then divided by IMR	Input-oriented DEA, Output-oriented DEA	24 OECD countries	2000
Bhat (2005) [4]	1. Inpatient Beds per 1000 population 2. Number of practicing physicians per 1000 population 3. Per Capita Pharmaceutical Expenditures	1. Percent of Population Aged 0–19 Years 2. Percent of Population Aged 20–65 Years 3. Percent of Population Aged 65+	Input-oriented DEA	24 OECD countries	1996
Gearhart (2016) [8]	1. Per Capita Healthcare Expenditures, $US Purchasing Power Parity 2. Educational Attainment (Years)	1. LE 2. ISR, where ISR is the inverse of IMR	Hyperbolic order-alpha estimator	27 OECD countries	1997–2006
González et al. (2010) [9]	1. Per Capita Healthcare Expenditures, $US Purchasing Power Parity 2. School Life Expectancy (Years)	1. Healthy LE 2. Disability Adjusted Life Years (DALY)	Value efficiency analysis (VEA), using Input-oriented DEA	165 countries, 4 income groups	2004
Grosskopf et al. (2006) [11]	1. Public Healthcare Expenditures, % of per Capita GDP 2. Private Healthcare Expenditures, % of per Capita GDP 3. Per Capita Gross Capital Formation 4. Per Capita Labor Force 5. Primary Education Enrollment Rate	1. LE 2. Under-5 survival rate (U5SR), where U5SR is the inverse of the childhood mortality rate (CMR) 3. Per Capita GDP	Output-oriented DEA	143 countries	1997
Kim and Kang (2014) [13]	1. Average Years of Schooling, Women Aged 15+ 2. Public Healthcare Expenditures, % of government expenditure on health care in 2007 U.S. dollars	1. LE 2. Under-5 survival rate (U5SR), where U5SR is the inverse of the childhood mortality rate (CMR)	Input-oriented DEA	170 countries, 4 income groups	2007
Retzlaff-Roberts et al. (2004) [15]	1. School Life Expectancy (Years) 2. Gini Coefficient 3. Tobacco Use (Maximum of the percent of Male or Female Smokers) 4. Practicing Physicians per 1000 Population 5. Inpatient Beds per 1000 Population 6. MRI Units per 1,000,000 Population 7. Per Capita Healthcare Expenditures as Fraction of Per Capita GDP, $US Purchasing Power Parity	1. Life Expectancy 2. Infant Mortality Rate	Input-oriented DEA, Output-oriented DEA	27 OECD countries	1998
Spinks and Hollingsworth (2005, 2009) [22, 23]	1. School Life Expectancy (Years) 2. Unemployment Rate 3. Per Capita GDP 4. Per Capita Healthcare Expenditures, $US Purchasing Power Parity	1. LE	Output-oriented DEA	28 OECD countries	1995, 2000

In fact, these issues have led to a number of authors theorizing that second-stage estimation procedures are necessary to derive any inference from the many efficiency rankings studies. These second-stage estimation procedures would regress the efficiency rankings on environmental variables that impact health but that are largely outside of the control of healthcare authorities. Simar and Wilson [21], however, note that most of these second-stage regression papers ignore sizable theoretical issues with interpreting the results. They also note that standard ordinary least squares (OLS) estimates are only appropriate with extremely restrictive assumptions, and that bootstrapping is the only method to derive inference consistently.

This paper attempts to shed light on the problems inherent in the cross-country healthcare efficiency literature. It attempts to answer the question if there is considerable variation among efficiency rankings from the many input-output specifications provided by the 9 papers listed in Table 1. With different measures being utilized, there can be little consensus and little policy improvement in this sphere. It also asks whether the broad measures of healthcare efficiency (some measure of life expectancy, early age mortality, per capita healthcare spending, and total educational attainment) provide efficiency rankings that are more consistent across different specifications than the efficiency rankings found in more specific measures (healthcare utilization, population composition, composition (i.e., public or private) of healthcare spending). This would highlight the notion that many of the specifications from other studies including these more specific measures should use their measures in a second-stage estimation procedure, where these variables should be used as environmental variables. Not only may these broader measures be more appropriate, they are more likely to be collected on a consistent basis.

This paper finds that the lack of a theoretical input-output combination can lead to highly divergent efficiency rankings across specifications, and that if these specifications are being used to guide policy, policymakers can specification search for their preferred finding. In fact, this highlights the difficulty in using any of the literature in any policy-making manner.

This paper finds that efficiency studies that utilize the broad measures only ([8]; [9]; [22, 23]) have efficiency rankings that are strongly positively correlated with each other, and that studies that utilize alternative and more specific input and output measures have efficiency rankings that are weakly positively correlated (and sometimes negatively correlated) with each other. This shows the fact that other, important, socio-economic, quality, and demographic variables should be used in a second stage regression as environmental variables. It also finds that quality-adjusted output measures lead to efficiency rankings that are strongly positively correlated with efficiency rankings derived from output measures that do not include healthcare quality.

The paper is organized as follows: Section II details the data and estimators used, Section III reports the results, and Section IV concludes.

## Data and estimators

### Data

Data for this paper comes from the OECD Health Statistics database, as well as OECD statistics database. All available measures were calculated for the 30 OECD countries from 2000 to 2012. If data were not available for that year for a country, that country was indicated as having a missing value for that measure. All measures were altered by the author to conform with the data alterations conducted by the original papers. The data is available in Excel form upon request from the author. The variables used in the analysis for this paper are summarized in Table 1.

### Output quality measures

The original efficiency literature, utilizing cross-country comparisons, by Evans et al. [7] and Tandon et al. [24], attempted to incorporate output quality in a number of ways. The OECD has, at times, created a number of measures, such as disability-adjusted life expectancy (DALE), quality-adjusted life years (QALY), or disability adjusted life years (DALY) to take into account differences in the quality of outcomes. Alternatively, the Evans et al. [7] and Tandon et al. [24] studies attempted to mitigate the quality issues by creating a comprehensive output measure (termed COMP) that weighted different health goals, such as timeliness of care, differently.

Unfortunately, these quality-adjusted output measures are not as robust as they need to be when conducting efficiency analysis. Williams [27] and Richardson et al. [16] detailed the quite significant issues with the arbitrary weighting schemes utilized by Evans et al. [7] and Tandon et al. [24] when creating their COMP output measure. Hollingsworth and Wildman [12] noted that these quality output measures are often self-reported measures or utility measures (QALY). They noted that these measures are not used often because they are not routinely collected by various agencies, are subject to large variations in methodological rigor between countries, and are open to interpretation, which may violate the homogeneity assumption utilized by non-parametric frontier estimators.

Gearhart [8] noted that efficiency results were similar when utilizing DALY or life expectancy, so for methodological simplicity, life expectancy was chosen as the output measure. In this paper, quality measures are explicitly considered by two papers: (1) Adams et al. [1], where they modify infant survival rates and life expectancy by the ratio of the public spending on healthcare to total spending on healthcare (both as a percent of GDP), and (2) González et al. [9], who utilize both healthy life expectancy and DALY as their output measures. The rest of the papers summarized use unconditional output measures that do not take quality into account. The results of the relationship between papers with and without modified quality output measures will be discussed in the Results and Discussion section.

## Methods

### Limitations and benefits of different non-parametric estimators

Table 1 summarizes the estimators utilized by the 9 different papers under investigation. All papers in the sample utilize non-parametric estimators, rather than parametric estimators. Most of the papers utilize non-parametric data envelopment analysis (DEA) estimators. Only one paper [8] utilizes the order-α estimator that will be utilized to estimate all efficiency results in this paper. In this section, I describe the benefits of using non-parametric estimators in general, as well as the limitations of using the DEA estimators. I then describe why the order-α estimator seems to be superior, and then describe the calculations undertaken by the order-α estimator.

Non-parametric estimators are often used by researchers because they do not require an a priori specification of the functional relationship that is being estimated. Similarly, because of the lack of distributional assumption, incorporating multiple inputs or outputs is seamless. However, the DEA estimator used by many researchers suffers from well-known problems that make validity and inference a problem. The problems include the DEA estimator having less than root-n convergence due to the curse of dimensionality, where the number of observations required to obtain meaningful estimates increases with the number of production inputs and outputs used in the estimation; it also includes the estimator being sensitive to outliers [14]. The DEA estimator also, by construction, leads to many units in the analysis being considered fully efficient, as it is a full frontier estimator [20].

Alternatives to DEA estimators, such as the order- α estimator (which involves estimating a partial frontier lying “close” to the true production frontier), have been developed which alleviate many of these problems.^1^ Unlike the DEA estimator, the order-α estimator is robust to outliers. The order- α estimator is a partial frontier estimator, and it allows some observations to lie above the estimated partial frontier, limiting the impacts of extreme values (or outliers) on efficiency scores [20]. By design, this also limits the number of units that are deemed as fully efficient. The order-α estimator also addresses the curse of dimensionality found in DEA estimators; by design, the order-α estimator achieves the classical, parametric root- n rate of convergence, even though it is a fully non-parametric estimator [26]. The hyperbolic order-α estimator thus provides the distributional flexibility of non-parametric estimators while simultaneously providing traditional statistical features found in parametric estimators.^2^

### Hyperbolic order-α estimator methodology

Unfortunately, having to choose between an input-orientation (holding outputs fixed, can a country reduce healthcare inputs) and an output-orientation (holding inputs fixed, can a country increase healthcare outputs) leads to an issue surrounding the order- α estimator as well as the DEA estimator. In fact, Spinks and Hollingsworth [23] note that utilizing the input-orientation may not make sense for many health measures. As noted in Wheelock and Wilson [26], the choice between input- or output-orientation is often arbitrary. Wheelock and Wilson [25] offer a way out the choice between the input-orientation and the output-orientation. They describe an unconditional hyperbolic order- α quantile estimator that shares the advantages of the estimators described in Aragon et al. [3] and Daouia and Simar [5], but which avoids the third problem of choosing the orientation of the estimator. Since this paper is outside the context of a regression framework, the choice of direction function (input, output, or hyperbolic) does not have behavioral implications as it does in regression analysis; the hyperbolic distance function is therefore used. This allows for input contraction at a given output level, output expansion at a given input level, or a combination of input contraction and output expansion.

Due to this, the hyperbolic order- α estimator is utilized, which is a partial frontier estimator. The order-α estimator was developed a potential solution to the known problems in other non-parametric estimators described above, and where α ∈ (0,1] corresponds to the level of an appropriate non-standard conditional quantile frontier. The choice of α is continuous on the interval (0,1]. Wheelock and Wilson [25] define the hyperbolic order-α estimator as

1 $$ {\gamma}_{\alpha}\left(x,y\right)= sup\left\{\gamma >0\left|H\left({\gamma}^{-\mathsf{1}}x,\gamma y\right)>\left(\mathsf{1}-\alpha \right)\right.\right\} $$

using the Shephard [17] metric, where $H\left(x,y\right)= \Pr \left(X\le x,Y\ge y\right)$, which represents the probability that a unit operating at (*x, y*) is dominated (producing more output with the same level of inputs; producing the same level of output with less inputs; or producing more outputs using less inputs). *H* (*x, y*) is estimated by $\hat{H}\left(x,y\right)={\displaystyle {\sum}_{i=1}^n\left(\frac{I\left({X}_i\le x,{Y}_i\ge y\right)}{n}\right)}$, where $I\left(\cdot \right)$ represents the indicator function. *γ*_*α*_ is estimated by

2 $$ \hat{\gamma_{\alpha }}\left(x,y\right)= sup\left\{\gamma >0\left|\hat{H}\right.\left({\gamma}^{-\mathsf{1}}x,\gamma y\right)>\left(1-\alpha \right)\right\} $$

Wheelock and Wilson [25] establish the consistency of the hyperbolic order-α estimator.

If ${\gamma}_{\alpha}\left(x,y\right)=\mathsf{1}$, the point is said to lie on the hyperbolic order-α quantile and is dominated by firms with a probability of (1−α) [20]. Another useful feature of the order-α estimator is that the estimator has an asymptotic normal distribution [25].

## Results and discussion

### Data homogeneity

An assumption used by non-parametric estimators is that the units being considered are homogeneous (also known as independence). A number of authors that have undergone cross-country efficiency analysis have noted that OECD countries are suitably homogeneous to employ non-parametric efficiency estimation models [8, 10, 12]. In his paper, Greene [10] notes that almost all of the inefficiency noise in his study comes from non-OECD countries. Kim and Kang [13] utilize World Bank income groups to stratify their countries to satisfy the homogeneity assumption for the DEA estimator: importantly, all the OECD countries fall into the same World Bank income group, “high income”.

Simar and Wilson [18] note that the assumption of homogeneous units, in non-parametric efficiency analysis, is reasonable in many situations. Simar and Wilson [19], however, described a heterogeneous bootstrapping procedure that does not utilize the assumption of homogeneity, though it comes at the cost of increased computational length. Wilson [28] notes a variety of simple, non-parametric tests for independence that occurs before efficiency analysis needs to be taken place. Using several of the tests proposed in Wilson [28], this paper fails to reject the null hypothesis of homogeneity.^3^ The analysis therefore utilizes the assumption that OECD countries are suitably homogeneous.

### Sampling difficulties

One issue that arises with the various specifications chosen by the various papers is that many of the variables chosen are not collected regularly by the OECD or the countries themselves. Table 2 shows that, in 2000 and 2012, only a handful of countries have data for all of the specifications provided by all 9 papers.^4^^,^^5^ In fact, in 2000, only 4 countries are common between all 9 papers: Denmark, Finland, France, and the United Kingdom.

**Table 2:** Countries with missing observations in 2000 or 2012, 9 cross-country healthcare comparison studies

Author	2000 countries missing	2012 countries missing
Adams et al. (2011) [1]	Hungary, South Korea, Mexico, Turkey	Canada, Chile, Hungary, Mexico, New Zealand, Turkey
Afonso & St. Aubyn (2005) [2]	Belgium, Chile, Iceland, Ireland, Italy, Japan, South Korea, Luxembourg, New Zealand, Norway, Poland, Portugal, Switzerland	Denmark, Finland, Germany, Greece, Netherlands, New Zealand, Sweden, Switzerland
Bhat (2005) [4]	Belgium, Chile, Hungary, Iceland, Ireland, Israel, Italy, Luxembourg, Mexico, New Zealand, Poland, Slovenia, Switzerland, Turkey	Australia, Canada, Denmark, Greece, Hungary, Israel, Japan, Mexico, Netherlands, New Zealand, Portugal, Turkey, United Kingdom
Gearhart (2016) [8]	South Korea	Australia, Canada, Chile, New Zealand
González et al. (2010) [9]	Norway	Australia, New Zealand, Norway
Grosskopf et al. (2006) [11]	Austria, Chile, Czech Republic, Hungary, Mexico, Slovakia, Turkey	Austria, Canada, Czech Republic, Greece, Hungary, Iceland, Israel, Italy, Luxembourg, Mexico, Slovakia, Turkey
Kim and Kang (2014) [13]	Hungary, Mexico, Turkey	All OECD Countries
Retzlaff-Roberts et al. (2004) [15]	Australia, Austria, Belgium, Canada, Chile, Czech Republic, Estonia, Germany, Greece, Iceland, Ireland, Italy, Japan, South Korea, Mexico, Netherlands, New Zealand, Norway, Portugal, Slovakia, Slovenia, Spain, Sweden, Switzerland, Turkey, United States	Australia, Austria, Belgium, Canada, Chile, Denmark, Germany, Greece, Hungary, Ireland, Luxembourg, Netherlands, New Zealand, Norway, Poland, Portugal, Slovakia, Spain, Sweden, United Kingdom
Spinks and Hollingsworth (2005, 2009) [22, 23]	Hungary, Mexico, Turkey	Hungary, Mexico, New Zealand, Turkey

In one case, school life-expectancy and healthy life expectancy, utilized by González et al. [9], are measured in 2000 and 2012 only. Some authors have attempted data interpolation [8, 10, 15], but this comes with its own caveats. This supports one of the main findings of the paper: that broad, regularly collected input and output measures (some measure of life expectancy, early age mortality, total education, and per capita healthcare spending) should be utilized, rather than specific measures that are collected sporadically.

As can be seen from Table 2, the papers with the broadest input-output measures [8, 9, 22, 23] share 29 out of the 34 OECD countries in common in 2000, while sharing 26 out of the 34 OECD countries in common in 2012.

### Efficiency results and discussion

A first finding is that because of the lack of a theoretically justified input-output combination, there exists quite considerable variation in country efficiency rankings across specifications. Table 3 presents efficiency rankings from the following 4 papers: (1) Afonso and St. Aubyn [2]; (2) Bhat [4]; (3) Gearhart [8]; and (4) Kim and Kang [13].

**Table 3:** Cross-country efficiency rankings in 2010 for 23 countries using common hyperbolic order-α estimator

	Afonso & St. Aubyn (2005) [2]	Bhat (2005) [4]	Gearhart (2016) [8]	Kim & Kang (2014) [13]
Austria	22	23	14	6
Belgium	13	16	15	15
Canada	7	4	22	19
Czech Republic	17	18	9	21
Estonia	10	5	1	17
Finland	15	13	12	4
France	18	17	16	10
Germany	20	15	21	18
Iceland	12	14	10	2
Israel	8	2	5	5
Japan	4	3	8	11
Korea	1	9	6	12
Luxembourg	9	11	17	3
New Zealand	14	1	18	20
Norway	23	12	20	14
Poland	2	6	2	8
Portugal	3	20	3	1
Slovak Republic	19	21	11	23
Slovenia	5	7	7	7
Spain	11	19	4	9
Sweden	6	8	19	13
Switzerland	21	22	13	16
US	16	10	23	22

This is estimated for the year 2010. These are the efficiency rankings, by country, in 2010, using the hyperbolic order-α estimator

In 2010, only 23 countries have observations for all 4 of these papers.^6^ The first result becomes apparent: The U.S., which has been deemed to have one of the most inefficient healthcare delivery systems in the world [7, 8] ranks anywhere from 10th (middle of the pack) to 23rd (dead last). Other countries, such as South Korea and New Zealand, exhibit a high degree of variability in their rankings as well.

These efficiency rankings are an improvement on the rankings found in the original papers. In Afonso and St. Aubyn [2], 8 out of the 23 countries tie for being the most efficient producers of healthcare; these include Canada, Japan, the United States, Spain, and Sweden. In this study, as can be seen in Table 3, the highest ranking of any of these countries is 4th (Japan) and 6th (Sweden). In fact, there is only a mild positive correlation between the rankings found in this paper and those in Afonso and St. Aubyn [2], with a Pearson correlation coefficient of 0.32. There is a negative correlation between the rankings found in this paper and those in Bhat [4].^7^

These highlight the two methodological issues in Afonso and St. Aubyn [2] and Bhat [4], among others that use DEA, that are rectified using the order- ∝ estimator: (1) the use of a DEA estimator with few observations and many variables, and (2) potential incorrect specifications as to what a healthcare delivery system should focus on.

**Fig. 1:**
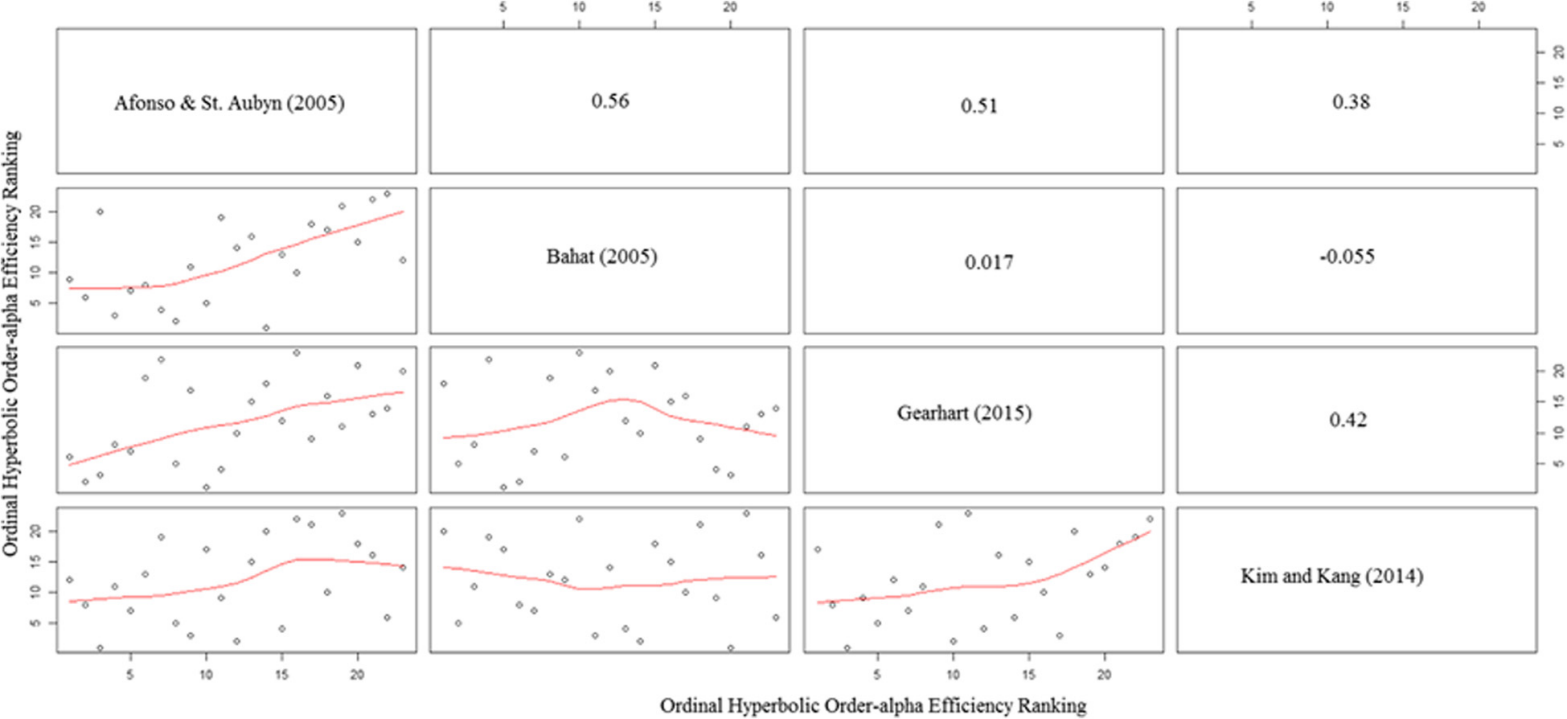
Cross-country efficiency ranking comparisons and Pearson correlation coefficients using hyperbolic order-α estimator, 2010. *NOTE: This figure compares cross-country efficiency rankings across a variety of paper specifications. Numbers in the upper triangle represent the Pearson correlation coefficient between any two sets of rankings

Figure 1 crosswise plots efficiency rankings from the 4 specifications in Table 3 with a line of best fit, as well as reporting the crosswise correlations between the papers. Afonso and St. Aubyn [2] have positive correlations with all other papers, ranging from 0.38 to 0.56. Most striking, however, is the fact that Bhat [4] varies significantly in how it correlates to the other papers. It has almost no correlation with Gearhart [8]. Bhat [4] is even slightly negative correlated with Kim and Kang [13], with a Pearson correlation coefficient of −0.06. This highlights the fact that even looking at a small number of studies that attempt to measure healthcare efficiency can lead to highly divergent efficiency rankings. One paper can find that a certain country is highly efficient in producing healthcare while another can find that the same country is highly inefficient. These findings have been supported in Gearhart [8].

This means that the lack of theoretical underpinning of these models can lead to policymaker specification searching, where by changing the input-output mix, they can obtain whichever answer they seek.

From Table 2, we note that broad input-output variables (some measure of life expectancy, early age mortality, total education, and per capita healthcare spending) are superior to more specific input-output variables, as they are collected more frequently, and allow for the analysis of efficiency gains over longer periods of time.^8^ This paper now attempts to support this finding from efficiency analysis, using results from a hyperbolic order-α estimator.

Table 4 shows results for the specifications provided in 6 papers in 2000.^9^

**Table 4:** Cross-country efficiency rankings in 2000 for 25 countries using common hyperbolic order-α estimator

	Adams et al. (2011) [1]	Gearhart (2016) [8]	González et al. (2010) [9]	Kim & Kang (2014) [13]	Spinks & Hollingsworth (2005, 2009) [22, 23]	Grosskopf et al. (2006) [11]^a^	Grosskopf et al. (2006) [11]^b^	Grosskopf et al. (2006) [11]^c^
Australia	15	22	13	18	23	12	19	22
Belgium	14	15	22	14	25	18	14	16
Canada	20	23	14	16	20	19	15	18
Denmark	19	24	25	19	14	20	23	21
Estonia	6	1	1	24	1	1	2	2
Finland	3	8	15	1	24	4	18	9
France	21	16	16	12	22	22	20	23
Germany	22	17	17	17	19	23	22	24
Greece	16	4	4	3	12	17	8	11
Iceland	10	11	10	7	6	7	24	17
Ireland	5	20	21	23	10	2	4	4
Israel	11	10	9	6	15	10	6	8
Italy	12	9	8	4	21	15	10	13
Japan	1	7	3	11	8	8	16	12
Luxembourg	8	21	6	9	5	3	3	3
Netherlands	17	18	23	10	3	13	13	15
New Zealand	13	12	18	22	11	16	5	6
Poland	2	2	2	8	2	5	1	1
Portugal	23	3	12	5	4	24	25	25
Slovenia	18	6	7	20	7	21	9	7
Spain	4	5	5	13	16	11	17	20
Sweden	7	13	19	2	17	6	12	5
Switzerland	24	19	11	15	9	14	21	19
UK	9	14	20	21	13	9	7	10
US	25	25	24	25	18	25	11	14

This is estimated for the year 2000. These are the efficiency rankings, by country, in 2000, estimated using the hyperbolic order-α estimator

^a^Grosskopf et al. [11] refers to utilizing, as inputs, public healthcare expenditures and private healthcare expenditures, both as a percent of per capita GDP

^b^Grosskopf et al. [11] refers to utilizing, as inputs, public healthcare expenditures and private healthcare expenditures, both as a percent of per capita GDP; the per capita labor force; and per capita gross capital formation

^c^Grosskopf et al. [11] refers to utilizing, as inputs, public healthcare expenditures and private healthcare expenditures, both as a percent of per capita GDP; the per capita labor force; per capita gross capital formation; and the primary education enrollment rate

Again, we see that across the wide variety of specifications, there is considerable variation in the efficiency rankings. For instance, Finland ranks anywhere from 1st (most efficient) to 24th (second most inefficient) out of 25 countries. Similar variability can be seen in the U.S., which rankings 11th to 25th. Note, however, that the rankings provided by the papers that utilize broad input-output measures [8, 9, 22, 23] seem to be much more consistent with each other than the rankings from the other papers, which utilize more specific input-output variables. Note that the broad input-output measures are ones that have some measure of life expectancy, some measure of early age mortality, some measure of total education, and some measure of per capita healthcare spending. These are measured consistently across countries and collected most years, and are able to capture the individual impacts on the whole population. More specific measures include the composition of spending (whether it is public or private), healthcare utilization (number of doctors, nurses, and beds per capita), and population composition.^10^ These measures are not collected consistently, and are better utilized as environmental variables in a second-stage estimation procedure.

**Fig. 2:**
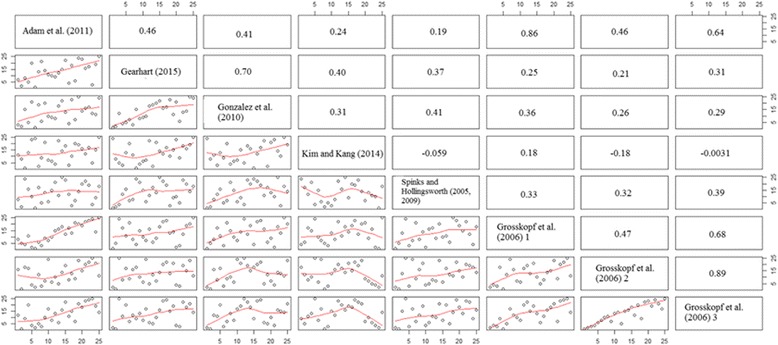
Cross-country efficiency ranking comparisons and Pearson correlation coefficients using hyperbolic order-α estimator, 2000. *NOTE: This figure compares cross-country efficiency rankings across a variety of paper specifications. Numbers in the upper triangle represent the Pearson correlation coefficient between any two sets of rankings

This can be seen better in Fig. 2, which plots the pairwise efficiency rankings and correlation coefficients from the papers found in Table 4.

In general, there is a positive correlation between most of the rankings in all of the papers. The average correlation is weakly positive, 0.36 (the median correlation being 0.34). However, nearly two-thirds of the correlations are less than 0.4 (weakly positive), while almost 10 % of the correlations are negative. The 3 papers that utilize the broad input-output measures [8, 9, 22, 23] have Pearson correlation coefficients that are higher, when compared to one another, than the other specifications that use more specific measures. The average Pearson correlation coefficient between the papers that utilize the broad measures [8, 9, 22, 23] is slightly above 0.5, while the average for the rest of the specifications is slightly less than 0.3.

Not only are the broad measures collected more consistently (and allow for more testing), but they are more consistent across different specifications. This allows a bit of freedom for researchers to modify these measures, without changing much of the underlying efficiency structure. The fact that the specifications with more specific input-output variables lead to highly variable efficiency rankings also indicates an important point: these variables should be used as environmental variables in a second-stage estimation procedure, as they play an important role in influencing the effectiveness of healthcare, but may be largely outside of the control of policymakers, and may be related to structural features of a country.

Table 5 presents efficiency rankings, in 2012, from 4 studies: (1) Adams et al. [1]; Gearhart (2) [8]; (3) González [9]; and (4) Spinks and Hollingsworth [22, 23].

**Table 5:** Cross-country efficiency rankings in 2012 for 26 countries using common hyperbolic order-α estimator

	Adams et al. (2011) [1]	Gearhart (2016) [8]	González et al. (2010) [9]	Spinks & Hollingsworth (2005, 2009) [22, 23]
Austria	25	18	18	9
Belgium	23	22	21	21
Czech Republic	7	9	4	5
Denmark	12	24	26	25
Estonia	3	1	1	2
Finland	13	14	22	22
France	21	19	12	19
Germany	22	21	23	16
Greece	11	4	13	13
Iceland	1	11	14	15
Ireland	10	20	19	24
Israel	6	7	3	10
Italy	14	10	10	11
Japan	8	12	7	4
Korea	5	6	5	1
Luxembourg	4	15	8	7
Netherlands	20	25	24	23
Poland	9	2	2	3
Portugal	15	3	15	12
Slovakia	16	8	6	8
Slovenia	2	13	20	6
Spain	19	5	9	17
Sweden	17	16	17	20
Switzerland	24	17	16	18
UK	18	23	11	14
US	26	26	25	26

This is estimated for the year 2012. These are the efficiency rankings, by country, in 2012, estimated using the hyperbolic order-α estimator

**Fig. 3:**
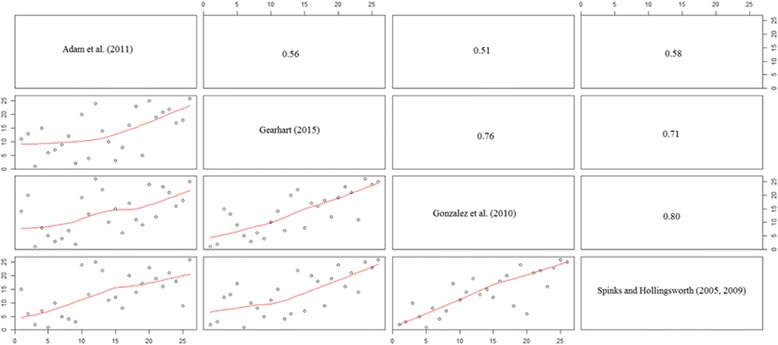
Cross-country efficiency ranking comparisons and Pearson correlation coefficients using hyperbolic order-α estimator, 2012. *NOTE: This figure compares cross-country efficiency rankings across a variety of paper specifications. Numbers in the upper triangle represent the Pearson correlation coefficient between any two sets of rankings

Adam et al. is similar to the latter 3 studies that utilize the broad measures of healthcare, but differs in a particular matter; it uses the composition of healthcare spending spending (public spending on healthcare as a percent of GDP) as an input, rather than per capita healthcare spending. This introduces difficulties into the estimation, as noted by Dyson et al. [6] and Gearhart [8]. This can be seen in Fig. 3, which pairwise compares efficiency rankings (as well as providing the Pearson correlation coefficient) between the rankings from the 4 studies.

As can be seen, the introduction of the composition of healthcare spending, rather than per capita healthcare spending, means that the efficiency rankings utilizing the specification from Adams et al. [1] are less consistent than the specifications in the other 3 specifications. This again supports the findings in Dyson et al. [6] and Gearhart [8] that including the composition of spending on healthcare can influence the results by perhaps eliminating the homogeneity of the countries under observation. This supports again the main findings: that broad measures of healthcare should be used as input-output variables, rather than more specific measures. Instead, these specific measures, because they do influence efficiency rankings quite considerably (when looking at the considerable variation in efficiency rankings, as seen in Fig. 2) should be utilized as environmental variables in a second-stage estimation procedure.

Figure 3 also highlights another main result in this paper: that utilizing more consistently collected output measures that do not adjust for healthcare quality (at the country level) seem to provide results that are highly consistent with those that use quality-adjusted output measures. Gearhart [8] uses life expectancy and infant survival rates, both of which do not take into account quality. González et al. [9], however, utilized healthy life expectancy and DALY as their output measures, both collected as a way to measure quality of a healthcare system at the country level. These two papers utilize the same input combination, allowing us to analyze whether the efficiency ranking results are highly different. As can be seen in Fig. 3, the Pearson correlation coefficient between the rankings using the specification in Gearhart [8] and González et al. [9] is highly positive, at 0.76. Comparing González et al. [9] to Spinks and Hollingsworth [22, 23], the correlation is 0.8.

This highlights that non-quality adjusted output measures seem to be an adequate substitute for quality-adjusted output measures that are collected infrequently (as mentioned earlier, healthy life expectancy has been measured for 2000 and 2012 only). Fortunately, health agencies across the world are taking note of the need to create quality-adjusted output measures to properly account for heterogeneity in patient populations. This means that the utilization of quality-adjusted output measures should be more standard in the future.

## Conclusion

It has been shown that there are considerable limitations with the variety of specifications utilized in cross-country healthcare efficiency comparisons since 2005. In a total of 9 studies replicated in this paper, there have been 8 unique specifications. One problem includes the fact that it is impossible to crosswise compare all specifications for any year between 2000 and 2012, due to many variables being collected infrequently. When able to compare certain specifications with one another, there is considerable deviation between efficiency rankings across specifications; in general, there is only a mildly positive correlation between specification efficiency rankings.

This indicates that utilizing broad measures is the appropriate procedure for healthcare efficiency rankings. These variables (some measure of life expectancy, early age mortality, total education, and per capita healthcare spending) are collected consistently. As shown in Using specific healthcare measures and the resulting variability in efficiency rankings hints that they are inappropriate for efficiency rankings directly, but should instead be utilized as socio-economic, quality, and demographic environmental variables in secondary regressions.

This means that there is considerable opportunity for specification searching on the part of researchers and policymakers. Policymakers can choose which input and output combinations yield the results (either highly efficient or highly inefficient) they desire.

A second limitation is that though these studies can conceivably be reconciled using a two-stage regression framework, where many of the variables used in studies (such as per capita labor force and the fraction of the population that is over the age of 65) could be used as environmental variables, there are limitations on which techniques can be used, which may require more consistent data than is available to allow for bootstrapping. Though these issues can be overcome, it does hint that papers studying this problem should be scrutinized severely.

Further research should focus on utilizing a second-stage regression framework to incorporate some of the alternative inputs as environmental variables. This may allow researchers to determine the outside factors that impact efficiency estimates and may resolve the dispute between which measures should be included in an efficiency analysis. Additional research should also continue to develop a theoretical underpinning for which input-output combinations are desirable, though this may change over time. This backing should take into consideration data limitations and cross-country collection issues.

Lastly, the fact that output measures that are not adjusted for quality (life expectancy) are consistent substitutes for quality-adjusted output measures (DALY, QALY) means that cross-country analysis can still be conducted, while the OECD continues to develop, maintain, and produce these quality-adjusted output measures on a more consistent basis. It hints that though the development of these measures is optimal, they are not a necessary condition for evaluating healthcare efficiency across countries.

## Footnotes

1 Aragon et al. [3] and Daouia and Simar [5] estimate input- and output-oriented conditional quantiles of order- α.

2 These statistical features are a lack of sensitivity to outliers as well as the root- n convergence rate.

3 Results are available upon request from the author.

4 Other years in between 2000 and 2010 show similar results. I utilize 2000 because that year helped to jump start the cross-country healthcare comparison literature and 2012 because it is the most comprehensive year with data on the OECD website.

5 In fact, Retzlaff-Roberts et al. [15] have 26 missing country observations in 2000 and 20 in 2012. Their specification, for this reason, will not be utilized in this paper.

6 If we were to add 2 additional papers, [11] and [22, 23], the number of OECD countries that would have observations across all specifications falls to 17. This is a relatively unsuitable number of observations for a cross-section. This results is available upon request from the author.

7 These findings are available upon request from the author.

8 This would be done utilizing a Malmquist Index, which is not utilized in this paper, but can be seen in others [8].

9 Note that Table 4 supports the first finding; across the wide variety of specifications, there is quite considerable efficiency ranking variation.

10 The composition of healthcare spending, whether it is public or private (in terms of GDP or total healthcare spending), is not mentioned as a “broad” indicator for a variety of reasons. A main reason is that the composition of spending has historical and political explanations that may wrongly influence efficiency estimates. Another is that the introduction of this variable may call into question several of the underlying assumptions that non-parametric efficiency estimators are based on [6].
